# Advanced Pre-Clinical Research Approaches and Models to Studying Pediatric Anesthetic Neurotoxicity

**DOI:** 10.3389/fneur.2012.00142

**Published:** 2012-10-17

**Authors:** Cheng Wang

**Affiliations:** ^1^Division of Neurotoxicology, National Center for Toxicological Research, United States Food and Drug AdministrationJefferson, AR, USA

**Keywords:** development, anesthesia, neurotoxicity, mechanism, imaging

## Abstract

Advances in pediatric and obstetric surgery have resulted in an increase in the duration and complexity of anesthetic procedures. A great deal of concern has recently arisen regarding the safety of anesthesia in infants and children. Because of obvious limitations, it is not possible to thoroughly explore the effects of anesthetic agents on neurons *in vivo* in human infants or children. However, the availability of some advanced pre-clinical research approaches and models, such as imaging technology both *in vitro* and *in vivo*, stem cells, and non-human primate experimental models, have provided potentially invaluable tools for examining the developmental effects of anesthetic agents. This review discusses the potential application of some sophisticated research approaches, e.g., calcium imaging, in stem cell-derived *in vitro* models, especially human embryonic neural stem cells, along with their capacity for proliferation and their potential for differentiation, to dissect relevant mechanisms underlying the etiology of the neurotoxicity associated with developmental exposures to anesthetic agents. Also, this review attempts to discuss several advantages for using the developing rhesus monkey model (*in vivo*), when combined with dynamic molecular imaging approaches, in addressing critical issues related to the topic of pediatric sedation/anesthesia. These include the relationships between anesthetic-induced neurotoxicity, dose response, time-course, and developmental stage at time of exposure (*in vivo* studies), serving to provide the most expeditious platform toward decreasing the uncertainty in extrapolating pre-clinical data to the human condition.

## Introduction

Anesthetic drugs have been used for many years in pediatric patients without clinical evidence of adverse central nervous system sequelae. It is known that the most frequently used general anesthetics have either NMDA-type glutamate receptor blocking or GABA receptor enhancing properties. There is mounting and convincing pre-clinical evidence in animal models that anesthetics in common clinical use are neurotoxic to the developing brain; and accumulated data (Choi, 1988; Ikonomidou et al., 1999; Jevtovic-Todorovic et al., 2003; Wang et al., 2005, 2006) indicate the involvement of NMDA-type glutamate receptors in the etiology of neurotoxic effects of anesthetic agents. The clinical relevance of anesthetic neurotoxicity is an urgent public health matter.

Recent advances in our understanding of stem cell biology and neuroscience have opened up new avenues of research for detecting anesthetic-induced neurotoxicity, dissecting underlying mechanisms, and developing potential protection/prevention strategies against anesthetic-induced neuronal injury. The application of stem cell biology/models toward understanding issues relevant to developmental neurotoxicology has the potential to advance our understanding of brain-related biological processes, including neuronal plasticity and toxicity (Keirstead et al., 2005; Li et al., 2005; Lamba et al., 2006; Lee et al., 2006; Kang et al., 2007). This review discusses several advantages and important issues for using stem cells as models and advanced research approaches, e.g., calcium imaging, for addressing critical issues related to the toxicity of pediatric anesthetics. These include the relationships between drug-induced neurotoxicity and how stem cells can be used as tools for dissecting out mechanisms underlying pharmacological and toxicological phenomena during development.

The general anesthesia drug products have been used for many years in pediatric patients without direct clinical evidence of adverse central nervous system sequelae. Data in support of a correlation between surgery and subsequent neuro-physiological changes has accumulated over years. The use of a non-human primate model to decrease the uncertainty in extrapolating pre-clinical data (Slikker et al., 2007; Zou et al., 2009a; Brambrink et al., 2010) to the human condition (e.g., peri-operative neurotoxicity) continues to garner considerable interest among anesthesiologists and toxicologists, with growing recognition to be anticipated from surgeons and neonatologists. A host of mechanistic studies have been completed or are underway which have been helpful in providing rationale for the overall concern over anesthetic and sedative-induced neurotoxicity. These studies have been and will be instrumental in teasing apart the causalities, refining hypotheses, developing alternative or protective measures, and suggesting clinical strategies for assessing the phenomena in children. Such studies have ranged from cell culture to histopathology, behavioral tests to molecular imaging (*in vivo*) studies – including non-human primate (Xie and Tanzi, 2006; Slikker et al., 2007; Rizzi et al., 2010; Paule et al., 2011). Because it is difficult or impossible to disentangle the effect of anesthesia *per se* from the effects of surgery or preexisting pathologies that necessitate surgery, it is essential to continue studies in non-human primates in order to obtain valuable information on the time-course and severity of observed deficits. It will also be necessary to determine whether the injured brain can recover with no or minimal loss of function, or whether the injured brain can be protected from sedative/anesthetic-induced injury by the co-administration of anti-oxidants or other agents. In addition, the development of PET/CT imaging systems provide the ability to collect dynamic, sensitive, and quantitative three-dimensional molecular information from the brains of living subjects including non-human primates and humans.

## Stem Cell-Derived Models (*in vitro*), Calcium Imaging, and Underlying Mechanisms of Anesthetic-Induced Neurotoxicity

Advances in our understanding of stem cell biology and neuroscience have opened up new avenues of research for detecting early life stress-induced neurotoxicity and developing potential protection/prevention strategies against toxicant-induced neuronal injuries (Kiss et al., 1994; Wang et al., 1994, 1996; Brokhman et al., 2008; Trujillo et al., 2009). The classic definition of a stem cell requires that it has the capacity to self-renew and that it possesses potency. Self-renewal is defined as the ability of the stem cell to go through multiple cycles of cell division while maintaining its undifferentiated state (i.e., to generate daughter cells that are identical to it). Potency refers to the ability of the stem cell to differentiate into specialized cell types (Wilmut et al., 1997; Shamblott et al., 1998; Thomson et al., 1998). Stem cell biology, when exploited along with molecular signaling and biological approaches including calcium imaging, genomics, proteomics, and metabolomics, can be utilized to enhance our understanding of complex biological processes such as apoptosis, and can provide the fundamental concepts necessary for constructing models of the building blocks of biological systems during development. As these models evolve and become linked together as integrative modules, they provide the intermediate components necessary for use in a developmental toxicology approach/platform.

A neural stem cell is a subclass of precursor cells that has several specific characteristics: (1) self-renewing – capable of making additional copies of itself by division; (2) multipotent – capable of making daughter cells other than itself including committed progenitors, neurons, astrocytes, and oligodendrocytes; and (3) capable of generating all or part of neural tissue. Neural stem cells or neural progenitor cells are generally uncommitted and so can change their fate after exposure to salient environmental cues. Evidence shows that gene expression and the capacity for self-renewal and differentiation of neural stem cells are spatially and temporally specified. Thus, neural stem cells are defined as cells with the ability to proliferate, self-renew, and produce a large number of functional progeny that can differentiate into neurons, astrocytes, or oligodendrocytes and this characteristic, thought to be controlled by genomic, biochemical, and physical factors, is known as multipotency (Wang et al., 1996; Park et al., 2005; Brokhman et al., 2008).

The stem cell-derived models, especially human embryonic neural stem cells with their capacity for proliferation and potential for differentiation, have a great advantage for detecting potential anesthetic-induced neurotoxicity. This system provides a reliable, simple *in vitro* model, within a short time frame, for evaluating potential adverse effects and investigating the cellular mechanisms which may be associated with anesthetic-induced brain damage. Thus, stem cell-derived models should be one of the best systems in evaluating adverse effects of pediatric anesthetic exposure, because of: (1) source (some embryonic neural stem cells are directly from human fetuses); (2) specific cell types (the simplified *in vitro* system allows for examining adverse effects of anesthetics directly on neural stem cells, neurons, astrocytes, or oligodendrocytes; (3) using minimal numbers of animals in a short time frame; and (4) providing the opportunity for assessing the brain’s own regenerative capacity after experiencing events related to overdoses or prolonged exposures to drugs including some general pediatric anesthetics or environmental chemicals.

This review presents an overview of representative general anesthetics – primarily ketamine – as examples of how stem cells can be valuable in identifying the doses and time-course over which individual drugs produce damage and/or protect neural stem cells and cells derived from them, change their proliferation rate, and alter their fate (differentiation into neurons, oligodendrocytes, and astrocytes) *in vitro*. Here, a strategy of the use of embryonic neural stem cell cultures for monitoring potential adverse effects of ketamine on the neural stem cell expansion, proliferation, differentiation, and receptor function has been defined.

Ketamine, a non-competitive NMDA receptor antagonist, is a widely used general pediatric anesthetic agent. Lines of evidence have demonstrated that ketamine causes neuronal cell death in several major brain areas of experimental animals at an early developmental stage, e.g., during the brain growth spurt (Ikonomidou et al., 1999; Scallet et al., 2004; Slikker et al., 2007; Zou et al., 2009a). Apoptosis is a common cause of ketamine-induced neuronal cell death in rodents (Zou et al., 2009b). Previous works based on mRNA and protein levels have revealed that NMDA receptor NR1 expression in ketamine-treated rat pup brain was significantly higher than that in control rats (Slikker et al., 2007; Zou et al., 2009b; Shi et al., 2010). This is indicative of a compensatory up-regulation of NMDA receptors on the neurons, along with continued or prolonged NMDA receptor blockade by NMDA antagonists (e.g., ketamine). Previous evidence suggested that upon removal of ketamine from the extracellular milieu, the now up-regulated NMDA receptor population (compensatory regulation as a consequence of continued or prolonged NMDA receptor blockade) will “over” respond to normal levels of extracellular glutamate, resulting in glutamatergic excitotoxicity. However, several important questions remain unanswered.

NMDA receptors constitute a sub-family of glutamate receptors identified by specific molecular composition and pharmacological and functional properties. NMDA receptors are densely localized on neurons of most major brain areas and are physically connected to proteins involved in cell-signaling cascades (Arundine and Tymianski, 2003; Arundine et al., 2003). Glutamate is the primary excitatory neurotransmitter in the CNS of mammals. In addition to ionotropic receptors responsible for fast excitatory neurotransmission in the CNS, glutamate also activates a number of metabotropic glutamate (mGlu) receptors, which belong to the G-protein coupled receptor family of receptors. Glutamate stimulates the opening of the channels that the ionotropic receptors regulate to enable the influx of various ions and excessive activation of NMDA-type glutamate receptors is implicated in the pathophysiology of several neurological conditions including hypoxia-ischemia and seizure-mediated excitotoxic damage, neuropathic pain, and opiate dependence. Exploring the mechanisms by which anesthetic agents might disturb NMDA receptor expression patterns should help identify avenues for protection or prevention of potential anesthetic-induced neuronal damage.

Since NMDA receptors are highly calcium permeable, the interactions between altered ionotropic receptors (e.g., compensatory up-regulation of NMDA receptor) and intracellular calcium signaling [Ca^2+^]_i_, as well as how enhanced Ca^2+^ flux associated with ketamine exposure influences reactive oxygen species (ROS) generation and subsequent neuronal apoptosis, could appropriately be clarified, by monitoring changes in intracellular calcium concentration, e.g., Fura-2 AM live cell calcium imaging. Thus, the relationship between anesthetic (ketamine)-induced NMDA receptor dysregulation and signal transduction, could systematically be analyzed. Also, whether enhanced Ca^2+^ flux associated with up-regulated NMDA receptors (as a consequence of ketamine exposure) could increase ROS generation and subsequent neuronal apoptosis could be demonstrated.


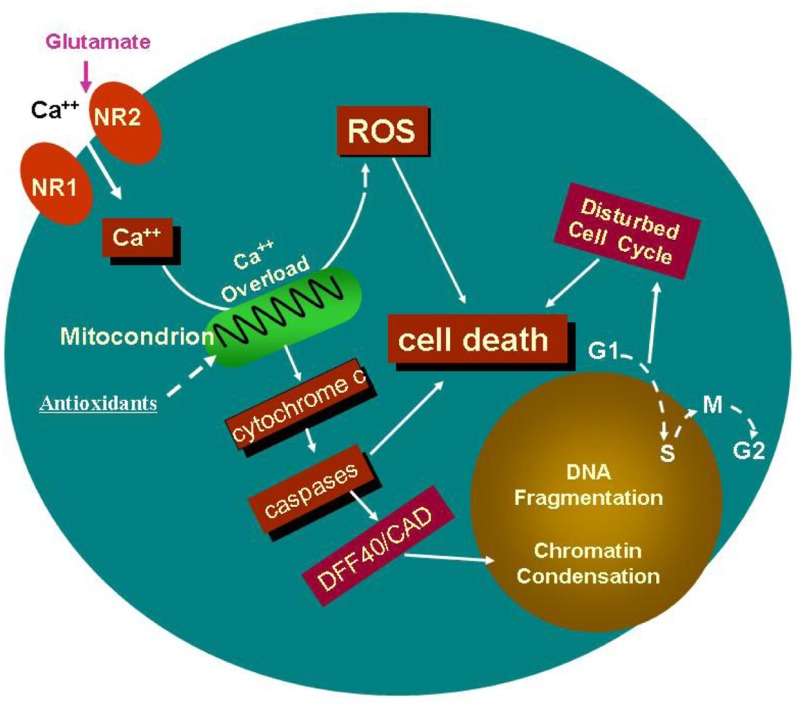


The cartoon (above) indicates a potential specific involvement of NMDA receptor-mediated excitation in ketamine-induced neurotoxicity. Continuous blockade of NMDA receptors by NMDA antagonists, such as ketamine, causes a compensatory up-regulation of the NMDA receptor. This regulation could make cells bearing the receptors more vulnerable, after ketamine washout, to glutamate, because this up-regulation allows for a toxic accumulation of intracellular calcium. Therefore, prolonged exposure of neural stem cells to ketamine results in intracellular Ca^2+^ overload that exceeds the buffering capacity of the mitochondria and interferes with electron transport in a manner that results in an elevated production of ROS. Studies *in vitro* and in intact cells have shown that caspase-3 specifically activates the endonuclease, CAD (Caspase-Activated Deoxyribonuclease). CAD then degrades chromosomal DNA within the nuclei and causes chromatin condensation. Also, ketamine may affect neural stem cell proliferation by slowing down, or even stopping the cell cycle, finally resulting in cell death.

Taken together, the use of neural stem cell models, especially those of human origin, when combined with calcium imaging and molecular biology approaches, holds promise for helping to elucidate relevant mechanisms underlying the etiology of the neurotoxicity associated with developmental exposures to the general anesthetics, and may also help identify avenues of protection or prevention. Data/observations related to NMDA receptor expression and function could provide further support to the idea, that in addition to NMDA-type glutamate receptor expression levels, the specific signal transduction (e.g., Ca^2+^ influx) plays a critical role in anesthetic (ketamine)-induced neurotoxicity.

## Application of Non-Human Primate Models (*in vivo*) and Dynamic Molecular Imaging Approaches to Studying Anesthetic-Induced Neurotoxicity

Recently described developmental neurotoxic insults involve the apoptotic cell death of neurons in the rodent brain following developmental exposure to sedatives and general anesthetics, such as ketamine and some inhalation anesthetics (Jevtovic-Todorovic et al., 2003; Scallet et al., 2004; Shi et al., 2010). When these compounds are administered to the neonatal rat or mouse, a rapid and significant increase in apoptosis occurs in several brain regions (Young et al., 2005). Because of obvious concerns, it is not possible to thoroughly explore this kind of adverse anesthetic effect on neurons in human infants or children, nor is it possible to obtain relevant dose response or time-course data about the potential sedative/anesthetic-induced neuronal cell death and associated behavioral deficits in humans.

The non-human primate is an animal model that has proved invaluable for informing aspects of human physiology, pathology, pharmacology, toxicology, and systems biology. No other commonly used research animal has a functional fetal-placental unit, a propensity for single births and a fetal-to-maternal weight ratio comparable to that of humans. Due to the complexity of the primate brain, the monkey is often the animal of choice for neurotoxicology experiments and, given the protracted period of brain development, the monkey is arguably the very best model for studies of developmental neurotoxicity. The phenomenon of interest in the present discussion (anesthetic-induced neuronal cell death in the brain) has also been previously observed in the non-human primate, *Macaca mulatta* (Slikker et al., 2007; Zou et al., 2009a). Thus, the relevance of the sedative/anesthetic-induced neuronal cell death observed in rodent models to children is inferred because similar effects occur in the developing non-human primate.

The first report regarding neuronal cell death in non-human primates exposed perinatally to anesthetics was published in 2007 (Slikker et al., 2007). This study focused on the representative general anesthetic, ketamine (a non-competitive NMDA receptor antagonist), which was administered as an intravenous infusion at doses sufficient to produce a light surgical plane of anesthesia (Slikker et al., 2007). The neurotoxic effects of these ketamine exposures were examined several hours after the end of the infusions, based on the hypothesis that ketamine (prolonged exposure) induces an up-regulation of the NMDA receptor (compensatory), causing neurons to be more vulnerable to the excitotoxic effects of endogenous glutamate after ketamine has been cleared from the system. A 24-h ketamine infusion in postnatal day (PND) 5 monkeys was shown to produce a large increase in the number of darkly stained TUNEL-positive cells which exhibited the typical nuclear condensation and fragmentation indicative of enhanced apoptotic cell death. The TUNEL assay relies on the detection of fragmented DNA strands.

The degree to which the nervous system is resistant to neurotoxic insults is highly dependent upon the stage of development. Because the brain growth spurt in both human and non-human primates extends over a much longer time period than in the rat, matching the timing of a developmental event in humans and non-human primates is less problematic than matching the same between primates and rodents. In addition to PND 5 monkeys, ketamine-induced neuronal degeneration was assessed in gestational day (GD) 122 and PND 35 monkeys (Slikker et al., 2007). As seen in the PND 5 monkeys, GD 122 fetuses showed clear ketamine-induced neuronal cell damage, whereas PND 35 monkeys did not. GD 122 fetuses and PND 5 monkeys, thus, are more sensitive to ketamine-induced cell death than PND 35 monkeys, when less synaptogenesis is occurring. Although a complete understanding of neuronal cell sensitivity to ketamine in the primate is not possible from these few early studies, it is apparent that rhesus monkeys are sensitive during the last 25% of gestation (term is 165 days) to sometime before PND 35. Equating relative stages of development between human and animal models is critical for the extrapolation of safety assessment data. It is generally believed that the non-human primate fetus (especially that of the rhesus monkey) and the human fetus are more similar in stage of maturation at birth as compared to rats that are relatively immature at birth. For example, both humans and rhesus monkeys are born with their eyes open at birth, whereas newborn rat pups are not. At PND 7 the rat pup is more similar in maturation to a monkey late in gestation than to an infant monkey. According to a recent review (Clancy et al., 2007), the GD 123 monkey fetus is roughly equivalent to a GD 199 human fetus as determined by cortical development, and both are in the range of 75–80% of normal term. Also, NMDA receptor binding sites are present in the human fetal brain by GD 115, increase until GD 140–150, and then decrease slightly by GD 168–182 (Haberny et al., 2002) and the localization of NMDA receptors in monkey cortex is similar to that observed in humans (Huntley et al., 1997).

Recent research efforts employing imaging techniques have covered a wide spectrum ranging from basic insights into normal physiology and disease processes to drug development (Eckelman, 2003). Molecular imaging using PET/CT is a state-of-the-art modality capable of obtaining *in vivo* measurements of multiple biological processes in various organs (Massoud and Gambhir, 2003; Min and Gambhir, 2004). Therefore, imaging technology has great potential for advancing the understanding of brain-related biological processes, including neuronal plasticity, neuronal degeneration/regeneration, and neurotoxicity (Zhang et al., 2009, 2011). Exposure of the developing mammal to NMDA-type glutamate receptor antagonists (e.g., ketamine or phencyclidine) affects the endogenous NMDA receptor system, endogenous neural stem cell expansion, proliferation, and differentiation, and enhances neuronal cell death (Slikker et al., 2005). Based on previous data, it is expected that by using microPET/CT instrumentation long-lasting pathological changes in animals associated with general anesthetic exposure can be observed in living subjects (*in vivo*).

A growing body of data indicates that molecular imaging with isotope-labeled biomarkers (radiotracers) may help to detect neurotoxicity in infants, young, and adult animals (Zhang et al., 2009, 2011). The high-resolution positron emission tomography scanner (microPET) can provide *in vivo* molecular imaging at a sufficient resolution to resolve both major structures and neuronal activities in the non-human primate brain. To determine whether prolonged sedative/anesthetic exposure during development is associated with subsequent long-term cognitive deficits, anesthetic drug-induced neurodegeneration can be explored by monitoring changes in the uptake (binding) of radiotracers (e.g., [^18^F]-Peripheral Benzodiazepine Receptor ligand, a biomarker of neurotoxicity- and gliosis), in specific regions of interest in the monkey brain.

Meanwhile, studies are underway to employ radioactive tracers (PET) designed to target specific stem cell ligands so that their anatomical locations (CT) can be determined and monitored. In addressing critical questions about the relationships between anesthetic-induced neurotoxicity and developmental stage at time of exposure, these molecular imaging tools could be utilized to monitor, *in vivo*, endogenous neural stem cell activity following exposure to general anesthetics. Here, the radiotracer likely to be useful in monitoring endogenous neural stem cell proliferation is 3′-deoxy-3′-[^18^F]-fluoro-l-thymidine ([^18^F]-FLT), one of the most widely used radiotracers for imaging cell proliferation (Shields et al., 1998). The synthesis of [^18^F]-FLT has recently been described (Shields et al., 1998; Slikker et al., 2005). FLT, a thymidine analog, is taken up by cells and phosphorylated by thymidine kinase (TK) 1, leading to intracellular trapping. The phosphorylated FLT is trapped within cells without being incorporated into the cellular DNA because it lacks a 3′-hydroxyl. TK 1 is a cytosolic isozyme of TK and its activity closely parallels that of cellular proliferation (Bading and Shields, 2008). Therefore, the retention of [^18^F]-FLT within the cells represents an *in vivo* marker that can be used for the visualization of cell proliferation.

It has recently been reported that [^18^F]-FLT was used in conjunction with PET imaging for the non-invasive detection of endogenous neural stem cell proliferation in the normal and ischemic adult rat brain *in vivo* (Jacobs et al., 2007; Rueger et al., 2010). Thus, [^18^F]-FLT in conjunction with PET imaging should be applicable for use with both rodents and non-human primates following exposure to ketamine. If such experiments are successful, then similar studies should be possible with human infants and children. There are also other tracer choices that might be useful for visualizing endogenous neural stem cell proliferation. For example, it has recently been reported that ^11^C-labeled 4′-methyl-thiothymidine ([^11^C]-4DST) is a useful radiotracer for the *in vivo* PET imaging of cellular proliferation (Toyohara et al., 2006, 2008, 2011, 2012). The rationales for using this particular compound are that it is resistant to degradation by thymidine phosphorylase and is incorporated into DNA (Toyohara et al., 2011).

## Summary

It has been proposed that pediatric sedative/anesthesia-induced neurotoxicity depends on the amount (dose) given, the duration of the exposure, the route of administration, the receptor subtype activated, and the stage of the neural development at the time of exposure. These factors are important because they can help identify thresholds of exposure for producing neurotoxicity in the developing nervous system. There are yet many questions to answer before the findings of pediatric drug-induced neurotoxicity observed in animals can be related to effects in humans. Also, the threshold doses and exposure durations necessary for safe and effective treatment, as well as possible protective strategies must be determined.

Therefore, a thorough characterization of neural stem cells (*in vitro*) and an appropriate application of cellular/molecular/biochemical research approaches, including calcium imaging (Ca^2+^ influx), are crucial for understanding the cellular processes underlying the expression of, and sensitivity to, neurotoxicity. Such information will be needed in order to increase the likelihood of the clinical success of our attempts to develop effective rescue and prevention strategies.

In addition, the utilization of the developing non-human primate, *in vivo*, with dynamic molecular imaging approaches as applied to neurotoxicology should provide a framework on which information can be arranged in the form of biological models to be used for dissecting out mechanisms underlying toxicological phenomena associated with exposure to compounds of interest. Meanwhile, the relationships between anesthetic-induced neurotoxicity and endogenous neural stem cell activity following exposure to general anesthetics could be appropriately elucidated. Critical data can be obtained non-invasively, repeatedly, and quantitatively in the same subjects (Myers, 2001; Chatziioannou, 2002).

## Disclaimer

This document has been reviewed in accordance with United States Food and Drug Administration (FDA) policy and approved for publication. Approval does not signify that the contents necessarily reflect the position or opinions of the FDA. The findings and conclusions in this report are those of the author and do not necessarily represent the views of the FDA.

## Conflict of Interest Statement

The author declares that the research was conducted in the absence of any commercial or financial relationships that could be construed as a potential conflict of interest.
